# The Circadian Regulation of Sleep: Impact of a Functional ADA-Polymorphism and Its Association to Working Memory Improvements

**DOI:** 10.1371/journal.pone.0113734

**Published:** 2014-12-01

**Authors:** Carolin F. Reichert, Micheline Maire, Virginie Gabel, Marcel Hofstetter, Antoine U. Viola, Vitaliy Kolodyazhniy, Werner Strobel, Thomas Goetz, Valérie Bachmann, Hans-Peter Landolt, Christian Cajochen, Christina Schmidt

**Affiliations:** 1 Centre for Chronobiology, Psychiatric Hospital of the University of Basel, 4012, Basel, Switzerland; 2 Division of Clinical Psychology, Psychotherapy and Health Psychology, Institute for Psychology, University of Salzburg, 5020, Salzburg, Austria; 3 Respiratory Medicine, Department of Internal Medicine, University Hospital Basel, 4031, Basel, Switzerland; 4 Department of Psychiatry, Public Health Office, 60313, Frankfurt am Main, Germany; 5 Institute of Pharmacology and Toxicology, University of Zürich, 8057, Zürich, Switzerland; University of Oxford, United Kingdom

## Abstract

Sleep is regulated in a time-of-day dependent manner and profits working memory. However, the impact of the circadian timing system as well as contributions of specific sleep properties to this beneficial effect remains largely unexplored. Moreover, it is unclear to which extent inter-individual differences in sleep-wake regulation depend on circadian phase and modulate the association between sleep and working memory. Here, sleep electroencephalography (EEG) was recorded during a 40-h multiple nap protocol, and working memory performance was assessed by the n-back task 10 times before and after each scheduled nap sleep episode. Twenty-four participants were genotyped regarding a functional polymorphism in adenosine deaminase (rs73598374, 12 G/A-, 12 G/G-allele carriers), previously associated with differences in sleep-wake regulation. Our results indicate that genotype-driven differences in sleep depend on circadian phase: heterozygous participants were awake longer and slept less at the end of the biological day, while they exhibited longer non rapid eye movement (NREM) sleep and slow wave sleep concomitant with reduced power between 8–16 Hz at the end of the biological night. Slow wave sleep and NREM sleep delta EEG activity covaried positively with overall working memory performance, independent of circadian phase and genotype. Moreover, REM sleep duration benefitted working memory particularly when occurring in the early morning hours and specifically in heterozygous individuals. Even though based on a small sample size and thus requiring replication, our results suggest genotype-dependent differences in circadian sleep regulation. They further indicate that REM sleep, being under strong circadian control, boosts working memory performance according to genotype in a time-of-day dependent manner. Finally, our data provide first evidence that slow wave sleep and NREM sleep delta activity, majorly regulated by sleep homeostatic mechanisms, is linked to working memory independent of the timing of the sleep episode within the 24-h cycle.

## Introduction

The quantity and quality of sleep majorly depends on its timing. During the biological night (i.e., during phases of melatonin secretion), the human circadian pacemaker facilitates sleep initiation and preservation, while it actively promotes wakefulness during the biological day [1], [2]. Circadian wake promotion is paradoxically strongest at the end of a biological day [1], allowing the achievement of a consolidated wake period, despite homeostatic sleep pressure levels accumulating towards the end of the day [3]. In comparison, maximal circadian sleep propensity is observed in the early morning hours in order to prevent early awakenings, when sleep pressure has mostly dissipated during night-time sleep [4]. The combined action of circadian and sleep homeostatic mechanisms consequently allows the maintenance of sleep and wakefulness at appropriate times of the day [1], [3], [5].

Specific sleep features are differentially influenced by circadian and homeostatic mechanisms. For instance, while rapid eye movement (REM) sleep is strongly modulated by circadian phase [6], electroencephalographic (EEG) frequencies in the delta range are rather independent of time of day, but predominantly modulated by prior sleep time, mirroring sleep homeostatic processes [2]. Additionally, the overall regulation of the sleep-wake cycle by circadian and homeostatic factors exhibits large and stable inter-individual differences, which can partially be traced back to genetic variations such as the c.22G>A polymorphism (rs73598374) located in the gene encoding adenosine deaminase (ADA; [7], [8]). This polymorphism acts on sleep-wake regulation most likely through genotype-specific differences in the ADA-dependent metabolization of adenosine [9]–[13], which is involved in the regulation of sleep homeostasis [14]. Carriers of the G/A-allele, associated to a lower enzymatic activity of ADA [10], [13], show a higher homeostatic non rapid eye movement (NREM) sleep pressure, as indicated by higher night-time EEG activity in the slow wave and delta range, longer slow wave sleep (SWS) duration, and higher sleep efficiency [9], [12], [15], [16]. However, circadian contributions to the genotype-specific patterns in sleep structure and intensity remain unclear. Interestingly, we recently gathered first evidence that the circadian timing system varies according to the *ADA* polymorphism, since G/A-allele carriers exhibited a later onset of melatonin secretion [17], mirroring a shift in the opening of the gate for sleep [18].

Importantly, the dynamic interaction between homeostatic and circadian factors impacts not only on the timing of sleep and wakefulness, but also modulates a range of cognitive functions, among them working memory (WM) performance [19], [20]. The concept of WM refers to the temporary storage and manipulation of information. Previous investigations suggest improvements of executive aspects of WM performance, for instance in monitoring and manipulation of information held online, by training [21] as well as positive effects of night-time sleep [22], [23]. Moreover, we recently observed increased WM performance during a multiple nap compared to sleep deprivation protocol, specifically driven by heterozygous carriers of the *ADA* polymorphism [17]. However, it is unknown whether inter-individual differences in sleep-wake regulation can modulate the beneficial effect of sleep on WM and which specific sleep features contribute to sleep-dependent performance improvements. Also, it is unclear whether sleep-dependent benefits on WM depend on time of day, such that the advantageous effects occur only or most pronounced when sleep is expressed at a specific circadian phase, as shown for sequence learning and simple addition tasks [24], [25].

In the present investigation, a 40-h multiple nap protocol, similarly applied in prior studies (e.g., [26]–[28]), served to investigate circadian contributions under low sleep pressure levels to human sleep and waking functions with respect to the *ADA* polymorphism. We recently published data on behavioural effects of this genetic variation in response to different sleep pressure conditions (40-h sleep deprivation vs. the here reported 40 h of multiple napping). Working memory performance of G/A-allele carriers was more affected by sleep pressure manipulation than performance of G/G allele carriers. Here, we focus on the nap sleep protocol to investigate if characteristics over the circadian cycle are also differentially modulated by the *ADA* polymorphism, and whether they potentially associate to the reported genotype-dependent sensitivity to sleep pressure manipulation in working memory performance [17]. Concretely, we examined first if nap sleep, regularly scheduled along the circadian cycle, differs between G/A- and G/G-allele carriers under conditions of low sleep pressure. Sleep homeostatic and circadian mechanisms are inevitably linked such that a change of the state or dynamics on the one side entails a difference in the regulation in the other process (e.g., [19], [20], [29]). Considering the previously shown differences between genotypes in mainly homeostatic sleep features during night-time, we explored whether the circadian sleep-wake regulation might have adapted to these trait-like variations according to the *ADA* polymorphism. As circadian wake and sleep promotion is maximal at the end of the day and night, respectively, we assumed genotype-specific differences most likely to be detected during these crucial times of day. In a next step, we aimed at investigating the influence of nap sleep on WM performance, which was assessed before and after each of the scheduled naps. We explored which specific nap sleep properties act on WM performance and whether this is differentially expressed according to time of day and genotype. Based on prior evidence of a circadian modulation in the beneficial effect of sleep on cognition [24], [25], we hypothesized that sleep will boost WM performance in a time-of-day dependent manner, especially in case of sleep features being under strong circadian control (e.g., REM sleep duration).

Our data provide first evidence for a more distinct circadian modulation of nap sleep in G/A- compared to G/G-allele carriers. Further, WM performance benefits from REM sleep duration, observed particularly in the early morning during its circadian peak time, were more pronounced in heterozygous compared to homozygous individuals. In comparison, independent of time of day and genotype, WM performance improvements were positively associated to the amount of NREM delta power, a sleep feature mainly under sleep homeostatic control.

## Materials and Methods

### 1.1 Participants

As described earlier [17], 24 healthy young participants (12 G/A- and 12 G/G-allele carriers) out of 610 genotyped volunteers were willing to take part in the study. All participants were between 20 and 35 years old, healthy, non-smokers and free from depressive symptoms (Beck Depression Inventory [30], BDI-II <9). Exclusion criteria comprised transmeridian flights within three months before participation in the study, shift work, drug consumption or current medication (except contraceptives) and a history of prior psychiatric or sleep disorders. All participants slept habitually 8±1 h, stated a good subjective sleep quality (Pittsburgh Sleep Quality Index [31], PSQI≤5, see Table 1 for *M* and *SD* per genotype) and were medically screened by a physician before inclusion into the study. A screening night served to exclude sleep disorders and to habituate participants to the laboratory conditions. All women were tested for pregnancy before the laboratory part of the study and were required to participate during the luteal phase of their menstrual cycle (2 G/A- and 1 G/G-allele carriers) unless they were taking hormonal contraceptives. The genotype groups did not differ according to age, body mass index, subjective sleep quality, daytime sleepiness, chronotype and timing of sleep before and during study participation (*p*_all_>.10; for *M* and *SD* see Table 1).

**Table 1:** Demographic data and questionnaire scores (*M* and *SD*) split by genotype.

Sample characteristics	G/A-allele carriers	G/G-allele carriers	*p*
N (f, m)	12 (8, 4)	12 (8, 4)	1.00
Age (y)	24.33 (3.9)	24.75 (2.5)	.76
BMI (kg/m2)	21.80 (2.9)	21.60 (2.0)	.79
PSQI	3.58 (1.2)	2.83 (1.1)	.12
ESS	4.46 (2.8)	4.29 (2.0)	.87
MEQ	54.80 (9.7)	57.60 (10.8)	.51
MCTQ Sleep Duration	7.92 (0.6)	7.87 (0.7)	.82
MCTQ MSF sc	4.34 (1.1)	4.26 (1.0)	.84
MCTQ MSF sac	7.29 (2.4)	7.62 (2.7)	.75
Wake Time (hh:min) during study	07:08 (57 min)	07:13 (57 min)	.83

Notes. F =  female; m =  male; y =  years; BMI =  Body Mass Index, PSQI = Pittsburgh Sleep Quality Index, ESS =  Epworth Sleepiness Scale, MEQ =  Morningness-Eveningness Questionnaire, MCTQ =  Munich Chronotype Questionnaire, MSF sc =  Mid sleep free days sleep corrected, MSF sac =  Mid sleep free days sleep and age corrected. *P*-values were derived from χ2-(gender ratio) and t-tests (all other variables).

### 1.2 Genotyping

The procedure of genotyping has been described in detail in Reichert et al. (2014) [17].

### 1.3 Protocol and Procedure

The study was approved by the local ethics committee (Ethikkommission beider Basel) and performed according to the declaration of Helsinki. All participants gave written informed consent prior to study admission.

Before the laboratory part started, participants were asked to maintain a fixed sleep-wake cycle for one week (8 h±30 min time in bed during night-time, no naps allowed) in order to control for circadian misalignment and accumulation of sleep pressure during the week. Sleep-wake times were derived from a 3-week actimetry field study and if required, adapted to the participants' professional obligations. Actigraphical recordings served to verify compliance to the regimen. Furthermore, participants were instructed to abstain from alcohol and caffeine during this week in order to control for effects of these substances on sleep and waking functions [32]–[34].

**Figure 1:**
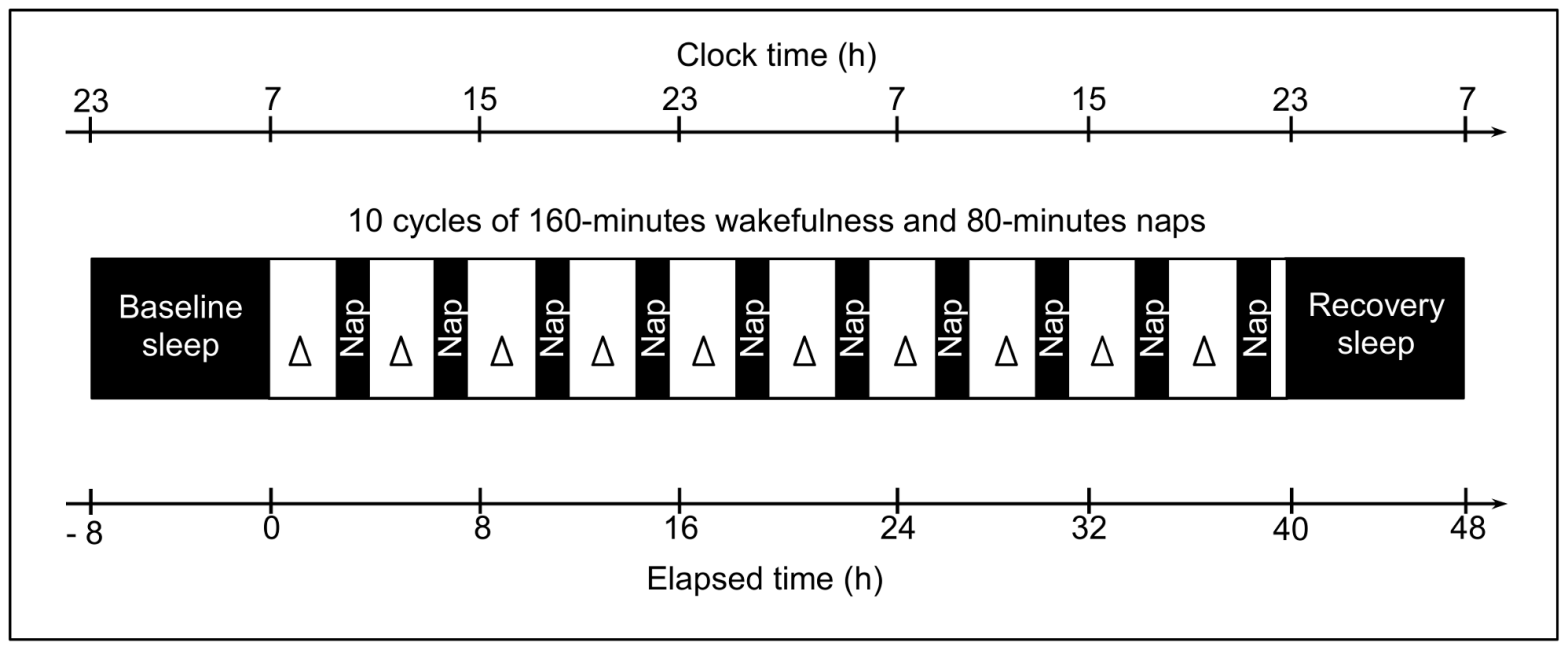
Schematic illustration of the laboratory part of the study. Following a 8-h baseline night, ten short sleep-wake cycles were scheduled over 40 h, each consisting of 160 min of wakefulness (white) under dim-light (<8 lux) and a 80-minutes nap (black bars, 0 lux). N-Back performance was assessed every 4 h (triangles) together with subjective effort, starting 1 h after waking up from the baseline night.

As reported previously [17], we implemented a randomized controlled within-subject design with two conditions, a nap and a sleep deprivation condition. Here, we mainly focus on data collected during the nap condition. The nap condition started with an 8-h baseline sleep episode. Following sleep, 120 min after regular wake-time, a repetitive short-day-cycle protocol started, with each cycle consisting of 160 min of wakefulness alternating with 80-min naps. After 40 h (encompassing 10 cycles), at regular bed time, the laboratory part ended with an 8-h recovery night (Figure 1). During wakefulness light was kept below 8 lux and body posture was semi-recumbent except for regularly scheduled bathroom visits. Meals were standardized and administered every 4 h (with a *SD* of 14 min). No indications of time of day were given. Social interaction was restricted to communication with study assistants. During scheduled sleep (at 0 lux), participants were asked to sleep if possible or to wait otherwise in darkness and recumbent position until the scheduled sleep episode has passed.

Beside the nap condition, a 40-h sleep deprivation was implemented in a randomized controlled order, separated by minimum 7 days from the nap condition. The sleep deprivation protocol was equal to the nap condition, except that no naps were scheduled [17]. Data of the sleep deprivation condition will be reported at the level of WM accuracy in order to verify that performance improvements occur specifically when participants were allowed to nap and do not solely reflect overall practice effects occurring with task repetition.

### 1.4 Melatonin

In order to determine circadian phase separately for each participant, salivary melatonin was collected with an average sampling rate of 60 min and analysed as previously reported [17]. Here, we focused on group comparisons of the dim-light melatonin onset (DLMO) and phase angle during the nap protocol. For definitions of DLMO and phase angle please see Reichert et al. (2014).

### 1.5 Nap Sleep

During the laboratory part of the study, polysomnographic signals (F3, FZ, F4, C3, CZ, C4, PZ, O1, Oz and O2 EEG derivations, two electrooculographic, two electromyographic and two electrocardiographic derivations) were recorded continuously with sintered MRI compatible Ag/AgCl ring electrodes with a 15 kOhm resistor (EasyCap GmbH, Germany) and V-Amp digital sleep recorders (Brain Products GmbH, Germany). All signals were sampled at 500 Hz and filtered online by applying a notch filter (50 Hz). Visual scoring of sleep stages was facilitated by filtering out frequencies below 0.1 Hz (high pass) and above 20 Hz (low pass) offline. Scoring of nap sleep was done according to standard criteria [35] by experienced staff blind to the genotype of the corresponding participant. Each file was scored by one scorer, and the number of files analysed by one scorer was balanced according to the genotype. Sleep latencies to stage 1, stage 2, and REM sleep were defined as time elapsed until the first occurrence of a respective epoch and analysed separately. All sleep latencies were log transformed before statistical analysis to achieve normal distribution. Slow wave sleep (SWS) was considered as sum of sleep stages 3 and 4, non rapid eye movement (NREM) sleep as sum of stages 2, 3, and 4. Sleep efficiency was calculated as percentage of total sleep time (TST, sum of sleep stages 1, 2, SWS and REM sleep) per nap. The timing of nap sleep was adjusted according to the DLMO of each participant and pooled to 4 h bins.

For consecutive 20-sec epochs, EEG power was calculated for artefact-free 4 sec epochs and averaged, using a fast Fourier transform with Hamming window. The resulting 0.25-Hz frequency resolution was analysed between 0.5 and 32 Hz. Here, we report EEG power spectra during NREM sleep (sleep stage 2, 3 and 4). A mixed model ANOVA with the factors genotype (G/A- and G/G-allele carriers), time (10 bins of 4 h) and hemisphere (left vs. right side derivations) did not reveal any significant interaction between genotype and hemisphere (*p*_all_>0.45). Thus, EEG spectra were collapsed along the anterior-posterior axis resulting in one value for each of the frontal, central, parietal, and occipital sites. In order to investigate the time course of sleep and wakefulness within the naps, specifically the distribution of SWS as an indicator for the dynamics of sleep pressure, nap-sleep was analysed per 20-min interval in each sleep episode. We particularly focused on the time course of sleep characteristics within the naps scheduled in the late evening (from 9:00 to 10:20 p.m. on average) and early morning (from 5:00 to 6:20 a.m. on average), encompassing maximal circadian drive for wakefulness and sleep respectively. As an indicator for sleep structure during the so-called wake-maintenance zone [5] in the late evening, a mean was computed of the two naps scheduled to 14 h and 38 h after regular wake-up time [26], [36]. This mean was compared with the nap starting 22 h after usual waking time in the early morning, that is, when the circadian sleep tendency is supposed to be strongest [1], [4].

### 1.6 N-back Task

Starting 60 min after waking up, participants were asked to perform a visual verbal n-back task, which was repeatedly administered every 4 h (i.e., 1 h before each nap), every other session in a magnetic resonance imaging scanner. The task lasted approximately 20 min and consisted of the visual presentation of 14 blocks of 30 consonants each (1.5 sec presentation time for each consonant, 0.5 sec inter-stimulus interval) on a computer screen. The volunteer's challenge in the n-back task is to decide and to indicate by a button press whether the letter presented is the same as n trials before. During each session subjects performed 9 blocks of 3-back and 5 blocks of 0-back-tests presented in a randomized order, each separated by a pause with a randomly generated duration of 10–20 sec during which a fixation cross was displayed on the screen. The order of the consonants per block and the number of targets per block was fixed (10 targets). The same block was not repeated within a session and appeared maximally 2 times over the course of the study, separated with at least 20 h in between. Performance was calculated by subtracting false alarms from hit targets (hit targets – false alarms) in order to measure the accuracy of the responses [37].

The n-back has been shown to be a useful measure specifically of executive aspects of WM as it requires permanent updating and manipulation of information [38], [39]. Other processes, such as inhibitory control, familiarity- and recognition-based discrimination and attentional processes are implicated in performance as well [40], [41]. In the 0-back, participants were asked to react to a specific consonant with a button press, such that they were not required to keep and manipulate information in WM, but still need to decide and to react correctly to the target stimulus. Following the methodology of subtraction [38], we report difference values (3-back – 0-back) of accuracy in order to account for basic attentional resources and inhibitory control, referring to this measure as WM accuracy. WM accuracy was adjusted according to the DLMO of each participant and collapsed into 4 h bins. For quantification of improvements from before to after sleep, difference values were calculated by subtracting WM accuracy values assessed before a nap from those acquired after the nap (after-before).

In the evening before the study, participants were trained in n-back-performance until they reached 70% of correct responses in the 3-back version of the task in order to prevent effects due to baseline differences in comprehension and transfer of instructions. Nonetheless, one heterozygous participant performed 3 interquartile ranges below the 25^th^ percentile during the entire course of the protocol. This performance was considered as an extreme value [42] and excluded from all analyses. Additionally, when quantifying performance changes from before to after a sleep opportunity in the late morning hours, the value of a homozygous participant was located 2 interquartile ranges above the 75^th^ percentile, and thus excluded as an extreme value for the respective analysis, too [42].

### 1.7 Subjective Effort

After each test bout, consisting of the n-back task followed by a 10-min vigilance test (modified version of the psychomotor vigilance task [43]), subjective effort was assessed by means of visual analogue scales. Participants were asked to indicate on three separate scales ranging each from 0 (*little*) to 100 (*much*) how much they had to endeavor and to concentrate during task performance, as well as to what extent the tasks were tiresome. Means calculated over these scales were adjusted to DLMO. In order to quantify changes in subjective effort from before to after sleep, values assessed before a nap were subtracted from those acquired after the nap (after-before).

### 1.8 Statistics

Statistical analyses were performed with SAS 9.3 software (SAS Institute, Cary, USA) using t-tests and mixed-model analyses of variance for repeated measures. T-tests for independent groups were used to evaluate differences between genotypes in the timing of melatonin. The general mixed model for analysis of sleep structure included the factor “genotype” (G/A-genotype and G/G-genotype), “time” (10 bins of 4 h), and “interval” (4 intervals of 20 min within each nap). The factor interval was not included for sleep latency analyses. For analysis of EEG power during NREM sleep, the mixed model for repeated measurements included the factors “genotype” (G/A-genotype and G/G-genotype), “time” (10 bins of 4 h) and “derivation” (frontal, central, parietal and occipital derivations). We did not include a factor “interval” due to a frequent lack of NREM sleep within the first two intervals of a nap (no NREM sleep at the beginning of a sleep episode due to wakefulness [50%] or REM sleep [5%]). If analysis of mean values in the delta (0.5–5 Hz), theta (5–8 Hz), alpha (8–12 Hz), sigma (12–16 Hz), beta (16–25 Hz) and gamma range (25–32 Hz) disclosed significant results, each frequency bin of the regarding frequency range was afterwards investigated separately. Analyses of WM accuracy and subjective effort included the factors “genotype” (G/A-genotype and G/G-genotype) and “time” (10 bins of 4 h). Contrasts of all mixed model analysis were calculated with the LSMEANS statement. Degrees of freedom of *p*-values are based on an approximation described by Kenward and Roger [44], and multiple post hoc comparisons were adjusted according to the Tukey-Kramer method [45]. *P*-values reported are adjusted for multiple testing.

The statistical software package SPSS 19.0.0 (IBM Corp., Armonk, USA) was used for analyses of covariance (ANCOVAs) to investigate both the influence of sleep features per se (SWS, REM sleep, SL1, NREM EEG delta, alpha and sigma activity) as well as the impact of these sleep features according to genotype (interaction genotype x sleep) on changes in WM accuracy within one statistical model. The difference in WM accuracy (3-back-0-back) between the first and the last test session in the study was considered as a global performance improvement index, independent of time of day. Genotype was considered as independent variable, sleep stages and intensities as covariates. Additionally, subjective effort to perform the task was included as a covariate into the model, since a recent study indicates that subjective effort influences n-back performance after sleep manipulation [46].

## Results

### 2.1 Melatonin

Mean values of DLMO and phase-angle (DLMO G/A-allele carriers 10:28 p.m.; DLMO G/G-allele carriers 09:43 p.m.; phase-angle G/A-allele carriers: 15 h and 20 min; phase-angle G/G-allele carriers: 14 h and 30 min) did not differ significantly between genotypes (DLMO: t_[11]_ = 1.76; *p* = 0.09; phase-angle: t_[11]_ = 1.89; *p* = 0.07), but yielded trend levels. Thus, as mentioned above, the timing of all repeated measurements was adjusted individually according to the DLMO of each participant.

### 2.2 Nap Sleep: Visual Scorings

**Figure 2:**
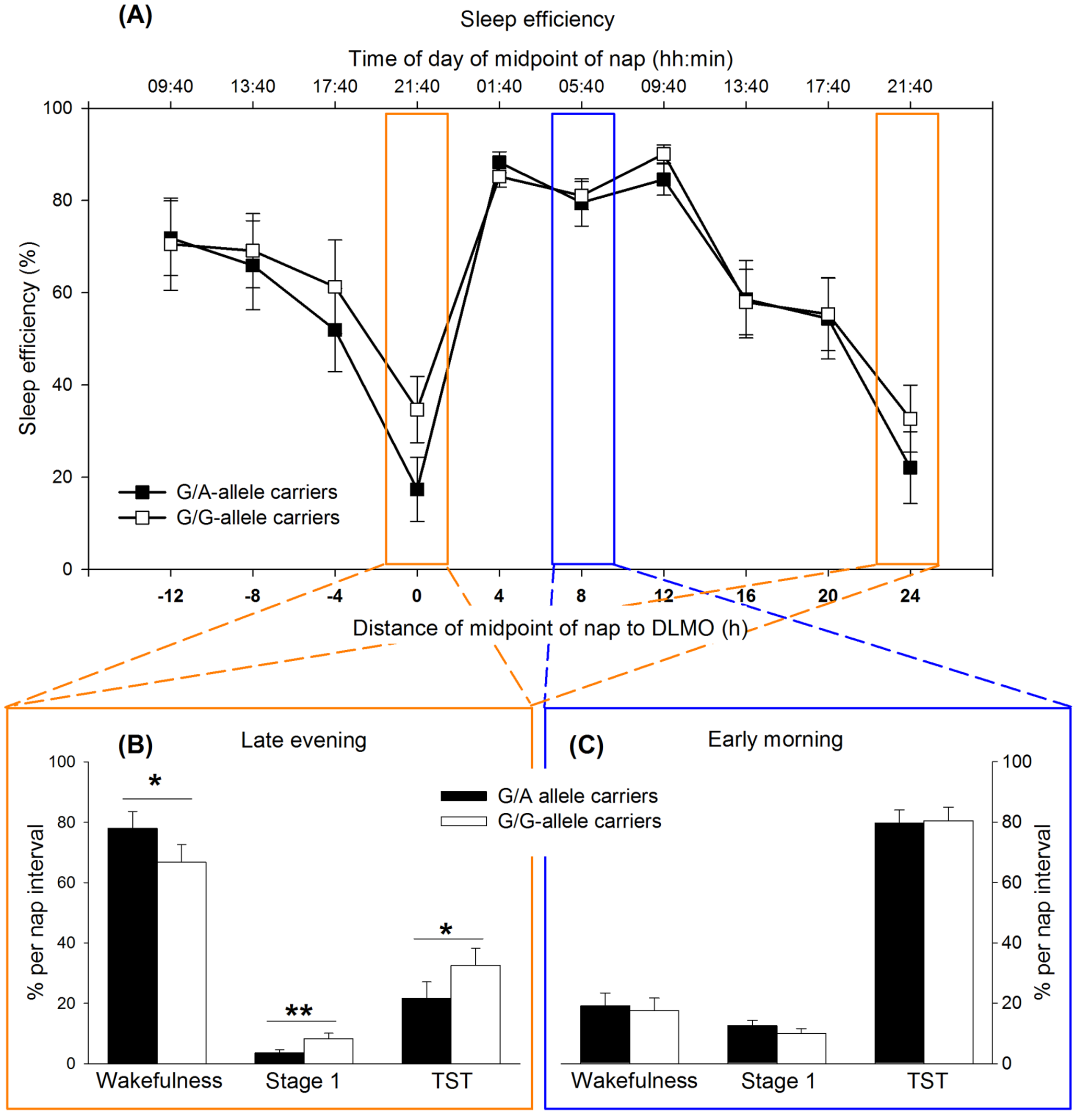
Sleep and wakefulness along the circadian cycle according to genotype. (A) Sleep efficiency was calculated by (sum of stage 1, 2, SWS and REM sleep)/(nap duration)*100. Sleep efficiency of G/A-allele carriers (black squares) and G/G-allele carriers (white squares) displayed a similar circadian pattern with low values in the late evening hours and high values during the biological night. (B) In the late evening hours, during highest circadian wake drive, the duration of wakefulness, stage 1 and total sleep time (TST) was modulated by genotype, while (C) genotypes did not differ in these variables during highest circadian sleep promotion (early morning). * *p*<0.05; **p<0.01.

Results revealed that the proportion of wakefulness and of sleep stages per 80-min sleep opportunity varied as a function of the 24-h cycle (for wakefulness, stage 1, stage 2, SWS, REM sleep, NREM sleep, TST, sleep efficiency and movements Fs_[9,>844]_>2.0, *p*_all_<0.05; sleep latency to stage 1, to stage 2 and to REM sleep Fs_[9,>195]_>19.5, *p*_all_<0.001), exemplarily depicted for sleep efficiency in Figure 2A. Further, the occurrence of wakefulness and sleep stages depended significantly on time elapsed within a nap (interval) such that wakefulness and sleep stage 1 occurred more likely at the beginning, while deeper sleep stages, movements and REM sleep were more likely at the end of a nap (Fs_[3,842]_>5.6, *p*_all_<0.001). This pattern was modulated by circadian phase: Considering the first half of a nap, the duration of wakefulness increased over the course of the biological day and comparably dropped as soon as passing into the biological night (F_[27,842]_ = 2.4, *p*<0.01), while stage 1 showed a reverse pattern (F_[27,842]_ = 3.8, *p*<0.001). The increase of deeper sleep stages towards the end of the nap was most pronounced in the first half of the biological night (Fs_[27,842]_>2.5, *p*_all_<0.001), while REM sleep increased especially in the morning of the first experimental day (i.e., the day following baseline sleep) and during night-time (F_[27,842]_>3.0, *p*<0.001). Across all nap opportunities, genotype did not significantly impact on visual sleep scorings (*p*_all_>0.51 except for REM sleep F_[1,22.3]_ = 3.5, *p* = 0.08). However, a significant interaction between genotype and circadian phase in sleep stage 1 (F_[9,843]_ = 3.5; *p*<0.001) indicated that G/A-allele carriers showed a shorter duration of stage 1 sleep during the nap in the late evening (from 9:00 to 10:20 p.m.) close to the DLMO compared to G/G-homozygotes (*p* = 0.02; Figure 2B).

In a next analysis (see methods), we aimed at contrasting sleep structure assessed exclusively during maximal circadian drive for wakefulness and sleep respectively, that is during naps scheduled to the late evening (from 9:00 to 10:20 p.m.) and early morning hours (from 5:00 to 6:20 a.m.), respectively [1]. As expected, participants spent more time awake, initiated sleep later and slept correspondingly less during naps in the late evening compared to the nap in the early morning (for wakefulness, stage 1, stage 2, SWS, REM sleep, NREM sleep, TST, movements and sleep efficiency, Fs_[1,154]_>6.3, *p*_all_<0.05; for sleep latency to stage1, to stage 2 and to REM sleep Fs_[1,22]_>45.7, *p*_all_<0.001). Overall sleep occurred generally more likely at the end of a nap and was modulated by circadian phase, such that sleep appeared later within naps in the late evening compared to the early morning (for wakefulness, stage 2, SWS, REM sleep, NREM sleep, TST movements and sleep efficiency effects of interval Fs_[3,154]_>3.8, *p*_all_<0.05; for wakefulness, stage1, stage 2, SWS, REM sleep, NREM sleep, TST and sleep efficiency all effects time × interval Fs_[3,154]_>3.8, *p_all_*<0.05). Importantly, as depicted in Figure 2B and 2C, genotype-dependent influences on sleep structure were modulated by circadian phase (wakefulness F_[1,154]_ = 5.5; *p* = 0.02; stage 1 F_[1,154]_ = 10.8; *p* = 0.001; TST F_[1,154]_ = 5.6; *p* = 0.02; sleep efficiency F_[1,154]_ = 5.6; *p* = 0.02): In the late evening G/A-allele carriers spent more time awake (*p* = 0.04) and slept less (*p*_all_<0.05) compared to participants with the G/G-genotype. This was by trend mirrored in less NREM sleep of G/A- compared to G/G-allele carriers at the end of the nap in the late evening (*p* = 0.06), while the duration of NREM sleep (*p* = 0.03) and in particular SWS (*p* = 0.001) was longer in G/A-compared to G/G-allele carriers at the end of the nap in the early morning (interactions genotype × time × interval: NREM sleep: F_[3,154]_ = 2.7; *p*<0.05; SWS F_[3,154]_ = 3.1; *p* = 0.03).

### 2.3 Nap Sleep: Spectral Analysis

**Figure 3:**
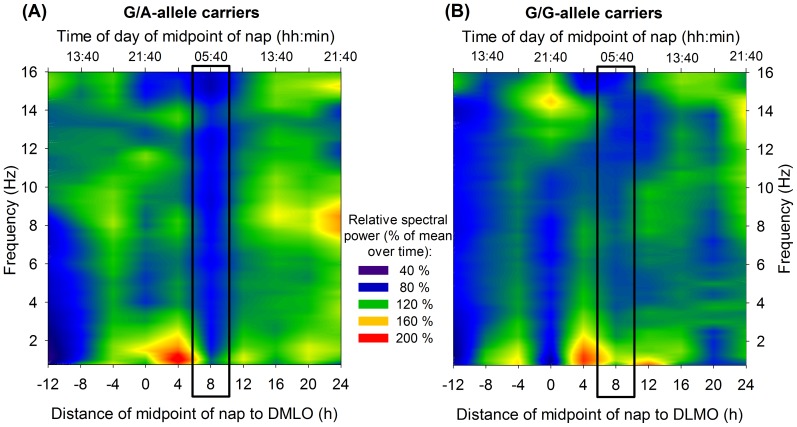
Relative EEG power density per genotype. Relative EEG power density is depicted as deviation from mean over time (i.e., over all naps) per genotype. Blue colours mirror relative decreases in EEG power density compared to the mean over time; green, yellow and red colours indicate relative increases of EEG power density compared to the mean over time. During the early morning hours (i.e., during the nap scheduled to 5:20–6:00 a.m.), highlighted by black boxes, G/A-allele carriers showed a relative decrease specifically in the range of 8–16 Hz (A), which was not present in G/G-allele carriers (B).

The well-known circadian phase and derivation-dependent modulations in EEG activity were evident over the entire power spectrum. The variations of EEG power along the circadian cycle are illustrated in Figure 3 as deviations from the mean over time per genotype. As depicted, a genotype-dependent impact on specific frequency bands became evident according to circadian phase (Table 2). Spectral EEG power in the delta range of G/G allele carriers (specifically between 0.5–2.5 Hz) dropped significantly *(p* = 0.003) from the early (5:00 to 6:20 p.m.) to the late evening hours (9:00 to 10:20 p.m.), and increased again (*p*<0.0001) when passing into the biological night (nap scheduled to 1:00 to 2:20 a.m.). This pattern was not present in G/A-allele carriers. Furthermore, EEG delta power (particularly in the range of 1.25–2.5 Hz) increased significantly (*p* = 0.01) from the early (5:00 to 6:20 p.m.) to the late morning (9:00 to 10:20 a.m.) in G/A-, but not in G/G-allele carriers (*p* = 0.99).

**Table 2:** Effects of genotype, time and derivation on delta, alpha, and sigma power.

Effect	Delta power	Alpha power	Sigma power
Genotype	F[1,22.3] = 0.2 *p* = 0.70	F[1,22.1] = 0.2 *p* = 0.72	F[1,22.1] = 2.5 *p* = 0.13
Time	**F[9,704] = 25.4 ** ***p*** **<0.0001**	**F[9,703] = 8.1 ** ***p*** **<0.0001**	**F[9,703] = 6.3 ** ***p*** **<0.0001**
Derivation	**F[3,702] = 198.8 ** ***p*** **<0.0001**	**F[3,702] = 120.8 ** ***p*** **<0.0001**	**F[3,702] = 327.0 ** ***p*** **<0.0001**
Genotype × time	**F[9,704] = 2.1 ** ***p*** ** = 0.03**	**F[9,703] = 2.4 ** ***p*** ** = 0.01**	**F[9,703] = 2.3 ** ***p*** ** = 0.01**
Genotype × derivation	F[3,702] = 0.6 *p* = 0.64	F[3,702] = 0.3 *p* = 0.88	**F[3,702] = 10.3 ** ***p*** **<0.0001**
Time × derivation	F[27,702] = 0.4 *p* = 1.0	F[27,702] = 1.0 *p* = 0.45	F[27,702] = 0.4 *p* = 1.0
Genotype × time × derivation	F[27,702] = 0.1 *p* = 1.0	F[27,702] = 0.2 *p* = 1.0	F[27,702] = 0.1 *p* = 1.0

Notes. Delta range: 0.5–5 Hz; alpha range: 8–12 Hz; sigma range: 12–16 Hz. F-values, degrees of freedom and *p*-values are derived from a ProcMixed ANOVA. Significant effects are printed in bold.

Dependent on circadian phase, genotype groups differed as well with regard to alpha power (Table 2, Figure 3). Only G/A-allele carriers showed a decrease in activity (*p* = 0.002), specifically between 8.5 and 12 Hz, in the early morning hours (assessed between 5:00 and 6:20 a.m.), which recovered afterwards in the late morning (9:00 to 10:20 a.m.; *p* = 0.0006).

Similarly, the influence of genotype on EEG power in the sigma range was modulated by circadian phase (Table 2; particularly between 12–12.75 Hz and 13.25–14.75 Hz) with a G/A-genotype-specific decline in the early morning (12–12.75; 13.25–13.75 Hz; 5:00 to 6:20 a.m.), followed by an increase during the late morning (12–12.25 Hz, 14.5–14.75 Hz; 9:00 to 10:20 a.m.). Additionally, analysis disclosed that the genotype-specific influence in the sigma power range (particularly between 11.75–16.5 Hz) differed according to derivation. However, post hoc comparisons did not reach significance after correction for multiple comparisons.

The EEG theta, beta and gamma activity did not significantly vary according to genotype (*p*_all_> 0.14).

### 2.4 N-back Performance and Subjective Effort

**Figure 4:**
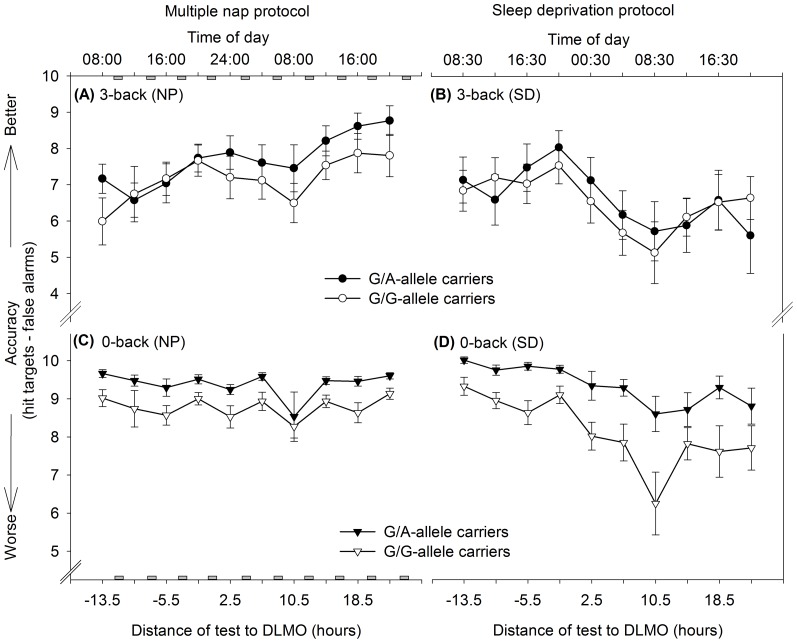
Accuracy patterns over time according to sleep pressure condition and genotype, separately for 3-back (upper panels) and 0-back (lower panels). Accuracy was calculated by a difference ratio (hit targets – false alarms). Grey rectangles indicate scheduled nap sleep episodes. In the 3-back task, accuracy improved from the first to the last test in the nap condition (NP, [A], F[9,183] = 11.66, *p*<0.0001; post hoc *p*<0.0001*)*, while the first and the last test did not significantly differ during sleep deprivation (SD, [B], F[9,184] = 8.84, *p*<0.0001, post hoc p>0.1). When working memory load was set to a minimum in the 0-back task (lower panels), accuracy remained stable from the first to the last test in the nap condition ([C], F[9,183] = 3.65, *p* = 0.0003; post hoc *p*>0.1), but decreased significantly during sleep deprivation ([D], F[9,183] = 3.65, *p* = 0.0003; post hoc *p* = 0.01). G/A-allele carriers performed constantly at a higher level in the 0-back version compared to G/G-allele carriers ([C], F[1], [21] = 8.17, *p* = 0.009), indicating differences in basic attentional resources between genotypes during the nap condition.

Figure 4 depicts the genotype-specific time courses of n-back accuracy throughout the 40-h nap protocol separately for 3- (Figure 4A) and 0-back (Figure 4C) in order to illustrate the evolution of accuracy under high compared to minimum working memory load. As mentioned (see methods), we calculated a difference ratio (3-back-0-back) of the depicted accuracy to account for variations in basic attentional resources and refer to this ratio as WM accuracy. WM accuracy values improved over time (F_[9,183]_ = 10.14; *p*<0.0001) similarly in both genotypes (genotype × time: F_[9,183]_ = 1.75; *p* = 0.08; post-hoc tests *p*_all_>0.6 after corrections for multiple comparisons): Participants performed significantly better during the last compared to the first session (*p*<0.0001). Importantly, such an increase in WM accuracy was not observed during 40 h of constant wakefulness neither under high (3-back, Figure 4B) nor minimum working memory load (0-back, Figure 4B). This result indicates that improvements in WM accuracy from the first to the last session were dependent on the reduction of sleep pressure by nap sleep and do not simply reflect general practice effects due to repetitive task administration. Sleep-dependent consolidation processes of working-memory related skills might associate to the benefits in WM accuracy observed after multiple napping.

Subjective effort brought up during task completion can influence n-back performance after sleep manipulation [46]. Thus, we investigated this measure in parallel to working memory accuracy. Subjective effort changed over time (F_[9,186]_ = 6.05; *p*<0.0001) exhibiting a circadian pattern: Participants perceived performance as less effortful during tasks scheduled in the evening (at 8:00 p.m.) of the first day compared to the tests scheduled before (*p*_all_<0.05). Afterwards subjective effort increased (*p*_all_<0.001) and stayed stable during the biological night. Starting around lunch time (12:00 a.m.) of the second day, participants indicated again task performance as less exhausting (*p*_all_<0.05). No genotype-dependent modulation was observed for this measure.

### 2.5 Relation between Nap Sleep, N-back Performance and Subjective Effort

In a final step, we explored whether the observed nap sleep-dependent improvements in WM accuracy from the first to the last test could be linked to specific sleep features collapsed over all circadian phases. We observed a positive impact of SWS (F_[1,7]_ = 11.46, *p* = 0.01) and NREM sleep EEG delta power (F_[1,7]_ = 17.28, *p* = 0.004) on WM accuracy improvements, such that a longer duration of SWS and a higher delta power was associated with greater WM accuracy benefits. Furthermore, the effect of REM sleep duration appeared to be modulated by genotype (F_[1,7]_ = 37.16, *p*<0.001), indicating a positive influence of REM sleep duration on WM accuracy improvements in G/A-, but not in G/G-allele carriers. Analyses of all other frequency bands and sleep stages did not indicate an association with WM accuracy improvements (*p*_all_>0.05).

**Figure 5:**
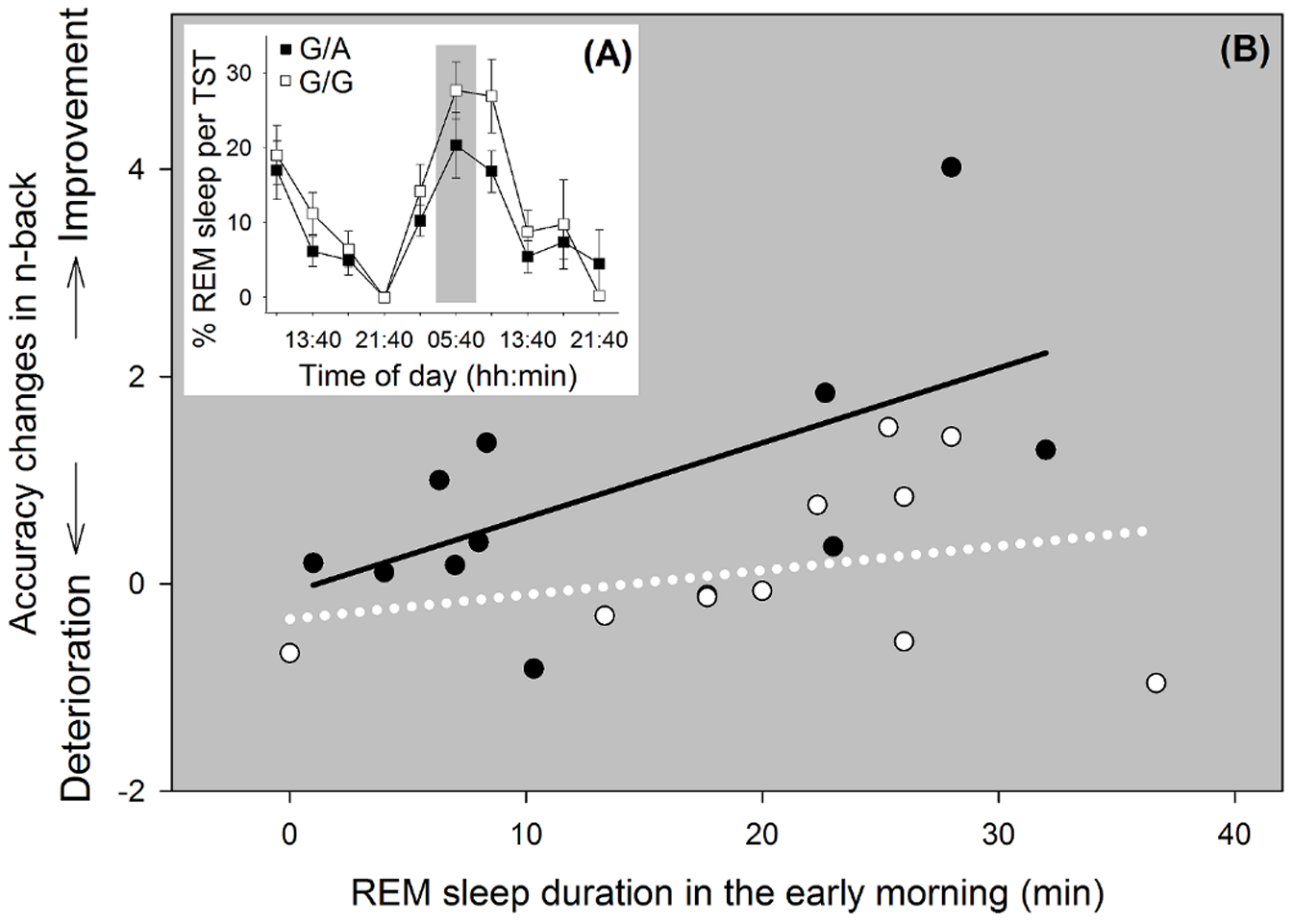
Association between REM sleep duration in the early morning and performance changes per genotype. (A) A strong circadian modulation in the proportion of REM sleep per nap was visible in both genotype groups (effect of time F[9,844] = 18.38, p<0.001; effect of genotype F[1,22.3] = 3.45, p = 0.08; interaction time × genotype F[9,844] = 0.96, p = 0.46) with a peak duration in the morning hours. The grey rectangle indicates the nap in the early morning (midpoint of nap: 5:40 a.m.), in which REM sleep duration was positively related to improvements in WM accuracy. (B) Performance changes are expressed as difference ratio (after nap sleep – before) in WM accuracy (3-back – 0-back). Overall, REM sleep duration in the early morning hours (5:20–6:00 a.m.) is positively related to improvements in WM accuracy (p = 0.02). The strength of this relation depends on genotype (p = 0.008) and is more pronounced in G/A-allele carriers (black solid line) compared to G/G-allele carriers (white dotted line).

In the light of the strong circadian regulation of REM sleep duration (e.g., [20]), we considered in a next step if the genotype-dependent impact of REM sleep duration on WM accuracy improvements is dependent on circadian phase. To do so, performance changes were quantified as difference ratios from before to after nap sleep episodes for those naps with a reliable REM sleep duration >5 min (mean of midpoints of excluded naps at the first day at 5:40 p.m. and 9:40 p.m., at the second day at 1:40 p.m. and 5:40 p.m.). For each of the remaining times of assessment, one ANCOVA was calculated aiming at a combined investigation of both the influence of REM sleep duration and its interaction with genotype on WM accuracy improvements, at the same time controlling for subjective effort. Results were adjusted for multiple comparisons according to the false discovery rate procedure [47]. This approach revealed that only REM sleep duration at the end of the biological night (5:00 a.m. to 6:20 a.m.) seems to affect subsequent WM improvement with a longer duration associated with higher performance increases (F_[1,18]_ = 6.3; *p* = 0.02; does not reach significance level when corrected for multiple comparisons). Importantly, this relationship was modulated by genotype (F_[1,18]_ = 9.0; *p* = 0.008) such that the beneficial effect of REM sleep duration on WM accuracy was more pronounced in G/A-allele carriers compared to G/G-allele carriers (Figure 5).

## Discussion

Our study suggests that the circadian regulation of sleep differs according to the *ADA* polymorphism, with the most prominent group differences during maximal circadian wake and sleep promotion. In parallel, results indicate that WM improvements depend on specific sleep characteristics. EEG delta power during NREM sleep as well as SWS was associated with WM accuracy independent of circadian phase and genotype. Positive effects of REM sleep duration appear particularly when it is expressed during its ‘natural’ circadian time window and seem to be more beneficial for G/A-allele carriers, presenting a more distinct circadian modulation in sleep structure and intensity.

### 3.1 Inter-individual differences in the circadian regulation of sleep

The *ADA* polymorphism has previously been associated with differences in sleep pressure levels, as indicated by genotype-dependent variations in subjective and behavioral variables as well as sleep during night-time [9], [12], [17]. The stronger behavioural vulnerability of G/A-allele carriers in response to sleep pressure manipulation we recently published [17] might at least partially be explained by the here observed differential circadian sleep regulation, as both sleep pressure and circadian processes tightly interact to produce consolidated sleep and wake bouts.

The wake-maintenance zone or ‘forbidden zone for sleep’ [48] reflects maximal circadian drive for wakefulness opposing high sleep pressure levels at the end of a regular waking day [1], [3]. At this time window, we detected higher amounts of wakefulness and shorter sleep duration in G/A-allele carriers, speaking in favour of a stronger circadian wake promotion in this genotype. The here observed more pronounced circadian arousal expression in heterozygous individuals might contribute to the previously reported improved working memory during multiple napping as compared to sleep deprivation [17]. The stronger wake promoting signal in the G/A-genotype might have evolved in order to oppose higher sleep pressure levels as reported under normal and high sleep pressure conditions [9], [12], [17]. Indeed, a differential circadian sleep-wake regulation according to the amount of accumulated sleep pressure has already been shown previously [19], [20], [29], [49]. Animal studies demonstrating a diurnal pattern of ADA activity in the rats' sleep-wake regulatory brain areas, such as the ventrolateral preoptic nucleus (VLPO) and the basal forebrain [50], suggest potential target sites at which ADA modulates circadian sleep-wake regulation. Note that it could be argued as well that genotype-dependent differences to initiate sleep at the end of the day may be attributed to a concomitant shift in the timing of melatonin [17], since the latter has been shown to play an important role in opening the gate for sleep [51], [52]. Nonetheless, by adjusting the analysis of sleep to DLMOs, we tried to control for this factor.

A genotype-specific pattern in the evening hours was as well observed in NREM sleep power in the low delta range. While G/A-allele carriers remained stable from the early to the late evening, G/G-allele carriers displayed a significant reduction in NREM sleep delta power during this time frame. At a first glance, this finding stays in contrast to the above discussed indications of a stronger circadian wake promotion in heterozygous individuals and seems to be in line with earlier reports of a higher SWA in G/A- compared to G/G-allele carriers [9], [12]. Note however that NREM EEG data could only be analysed from those participants who initiated NREM sleep during this time of strongest circadian wake promotion. Interestingly, these were by trend less G/A- (n = 4) than G/G-allele carriers (n = 9, χ^2^ test one-sided: *p* = 0.05). Considering the low sample size of G/A-allele carriers (n = 4), an interpretation at this level appears thus doubtful.

Besides promoting wakefulness during biological daytime, the circadian clock is also involved in sleep consolidation, which appears as particularly important in the early morning when sleep pressure has mostly dissipated under entrained conditions [1], [4], [53]. At the end of the nap in the early morning, particularly G/A-allele carriers maintained high levels of NREM sleep and SWS under low sleep pressure conditions. Concomitantly, they exhibited a pronounced decrease in sigma activity, which has previously been observed in recovery sleep after sleep deprivation [54], [55]. Beside sleep-homeostatic influences, sigma activity exhibits a strong circadian regulation [2]. In this perspective the data may point towards a trait-like, G/A-genotype-specific increased strength of the interplay between homeostatic and circadian sleep-wake regulatory factors.

Genotype-dependent differences at the end of the biological night were also detected in NREM sleep alpha activity. Alpha activity bursts during NREM sleep have been associated with cortical arousal [56]. Within this perspective, the G/A-genotype-specific alpha decrease suggests a genotype-dependent mechanism to prevent the interference of arousals for the achievement of a consolidated sleep period, even under conditions of low sleep pressure. However, together with a dominant vagal activity during NREM sleep, lower alpha activity has also been proposed to mirror processes of worse sleep maintenance during NREM sleep [57], such that the observed decrease in alpha activity of G/A-allele carriers at the end of the night might be associated to a decline in sleep maintenance. In the same perspective, alpha activity increases have been detected in recovery sleep following sleep deprivation [55]. Within this framework, a reduction of alpha power would paradoxically indicate a reduced sleep pressure in the G/A-genotype. Note however that in our protocol the state of the sleep homeostat was kept low by multiple naps. Under these conditions the homeostatic function of alpha activity [2] remains virtually unexplored.

By the implementation of multiple short sleep-wake cycles we aimed at specifically investigating the circadian regulation of sleep according to genotype under low sleep pressure conditions. Previous studies report genotype-dependent differences in SWS and SWA assessed during consolidated night-time sleep periods following intervals of 16 h [9], [12] or 40 h [9] of continuous wakefulness. However, by multiple napping we were able to assess the initiation of sleep as well as its structure and intensity in dependence of circadian phase. Sleep might be considered as a highly sensitive measure to unravel differences circadian sleep-wake regulation. Nonetheless, future studies should focus on the replication of genotype-specific differences in circadian regulation of sleep as similarly done for consolidated night-sleep episodes [15], [16].

### 3.2 Sleep-related ameliorations in WM performance

Previously, it has been shown that WM generally profits from sleep [23] and from low compared to high sleep pressure levels [17]. Here, we observed that both EEG NREM delta power and SWS promotes WM accuracy independent of circadian phase. NREM sleep delta power and SWS are conceptually linked and mirror mainly sleep homeostatic mechanisms [2], [20] while exhibiting a rather weak impact of circadian rhythmicity [2]. The homeostatic function of delta power has been linked to local modifications occurring at the synaptic level during cognitive challenges while awake [58]. In studies investigating the domain of visuomotor learning, enhanced delta power has been associated with prior mechanisms of encoding as well as with post-sleep benefits [59].

The circadian peak of REM sleep duration, mediated by the suprachiasmatic nuclei and their connections to orexin-containing neurons [60], occurs under entrained conditions in the early morning [6]. Our data indicate that REM sleep duration positively influences WM improvements, especially when occurring within this particular time. This highlights the impact of circadian processes on sleep-related cognitive performance modulation under low sleep pressure levels [24], [25], and suggests a possible circadian influence in the domain of working memory.

In the animal domain, Smith proposed a “paradoxical sleep window”, suggesting REM sleep to be specifically involved in memory formation (e.g., place learning or shuttle avoidance) at particular discrete time intervals [61]. The administration of cholinergic and dopaminergic antagonists [62] during such a window has been shown to impair memory formation. High cholinergic activity during REM sleep [63] has been associated with REM sleep-dependent memory consolidation in procedural learning [64]. In parallel, reduced acetylcholine levels in the prefrontal cortex impair WM performance [65]. In the light of changes in the dopaminergic system following WM performance trainings [66], the REM sleep-specific increase in dopaminergic activity [59] might additionally play a role in REM sleep-associated improvements of WM. This is supported by the observation that Parkinsonian patients under dopaminergic medication improved over night in WM span, compared to patients without dopaminergic medication [67].

Finally, our data reveal that the REM sleep benefits in WM performance is modulated by inter-individual differences in sleep regulation. This post-hoc observed result suggests that G/A-allele carriers appeared to be more sensitive for the association between REM sleep and WM performance. A higher sensitivity for circadian mechanisms underlying the beneficial effect of REM sleep on WM is plausible. Genotype-dependent differences in the adenosinergic system might impact on cholinergic and dopaminergic mechanisms [68], [69] potentially implicated in REM sleep-dependent benefits on WM performance.

Note that WM capacity, that is the maximum number of information that can be kept in WM, is classically considered as limited and fixed to a small number of items [70]. Improvements in n-back performance, as observed in the current study, do most probably not concern WM capacity, but reflect ameliorations in the executive aspects of WM. Such benefits in monitoring and manipulation of information held online as well as inhibition processes have been reported earlier [21] and might mirror changes in task-specific strategies. Whether these strategies can be generalized to other cognitive challenges of executive processes as reported for adaptive n-back task versions [21] remains to be elucidated.

This study is the first to demonstrate genotype-specific inter-individual differences in the circadian regulation of nap sleep and its association with working memory performance. However, regarding the sample size the result should be considered as preliminary as long as not being replicated by independent observations. Nonetheless, the data suggest the consideration of circadian mechanisms when investigating sleep-dependent performance improvements.
