# Correction: The Circadian Regulation of Sleep: Impact of a Functional ADA-Polymorphism and Its Association to Working Memory Improvements

**DOI:** 10.1371/journal.pone.0123502

**Published:** 2015-03-31

**Authors:** The PLOS ONE Staff

There is an error in Fig. 4, “Accuracy patterns over time according to sleep pressure condition and genotype, separately for 3-back (upper panels) and 0-back (lower panels).” Please see the complete, correct Fig. 4 and its legend here.

**Fig. 4:**
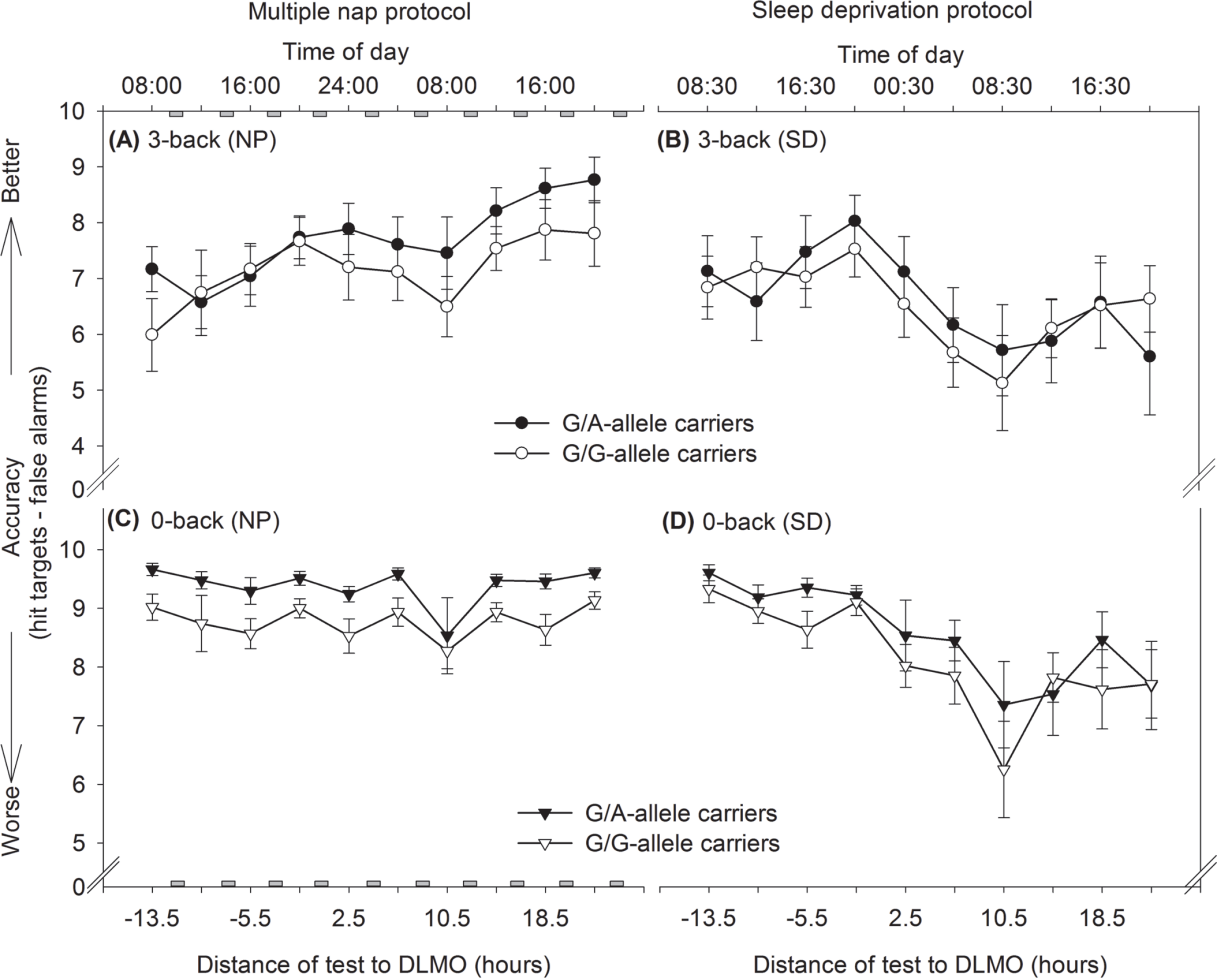
Accuracy patterns over time according to sleep pressure condition and genotype, separately for 3-back (upper panels) and 0-back (lower panels). Accuracy was calculated by a difference ratio (hit targets—false alarms). Grey rectangles indicate scheduled nap sleep episodes. In the 3-back task, accuracy improved from the first to the last test in the nap condition (NP, [A], F[9,183] = 11.66, *p*<0.0001; post hoc *p*<0.0001*)*, while the first and the last test did not significantly differ during sleep deprivation (SD, [B], F[9,184] = 8.84, *p*<0.0001, post hoc p>0.1). When working memory load was set to a minimum in the 0-back task (lower panels), accuracy remained stable from the first to the last test in the nap condition ([C], F[9,183] = 3.65, *p* = 0.0003; post hoc *p*>0.1), but decreased significantly during sleep deprivation ([D], F[9,184] = 8.62, p<0.0001; post hoc p = 0.01). G/A-allele carriers performed constantly at a higher level in the 0-back version compared to G/G-allele carriers ([C], F[1,21] = 8.17, *p* = 0.009), indicating differences in basic attentional resources between genotypes during the nap condition.
